# Update on Marine Carbohydrate Hydrolyzing Enzymes: Biotechnological Applications

**DOI:** 10.3390/molecules23040901

**Published:** 2018-04-13

**Authors:** Antonio Trincone

**Affiliations:** Istituto di Chimica Biomolecolare, Consiglio Nazionale delle Ricerche, Via Campi Flegrei, 34, 80078 Pozzuoli, Naples, Italy; antonio.trincone@icb.cnr.it; Tel.: +39-081-867-5095

**Keywords:** marine enzymes, biocatalysts, bioprocesses, glycosyl hydrolases, carbohydrate hydrolyzing enzymes, marine polysaccharides

## Abstract

After generating much interest in the past as an aid in solving structural problems for complex molecules such as polysaccharides, carbohydrate-hydrolyzing enzymes of marine origin still appear as interesting biocatalysts for a range of useful applications in strong interdisciplinary fields such as green chemistry and similar domains. The multifaceted fields in which these enzymes are of interest and the scarce number of original articles in literature prompted us to provide the specialized analysis here reported. General considerations from modern (2016–2017 interval time) review articles are at start of this manuscript; then it is subsequently organized in sections according to particular biopolymers and original research articles are discussed. Literature sources like the Science Direct database with an optimized W/in search, and the Espacenet patent database were used.

## 1. Introduction

Polysaccharides such as cellulose, starch, chitin, and many others constitute an important part of the general biomass. Science related to these molecules is of paramount importance for the life of humankind on our planet and biotechnological derived applications are of economic interest. Interdisciplinary topics related to these molecules include food and health in the nutritional/pharmaceutical fields, and many others. The inherent chemical structure complexity of carbohydrate molecules, greater than that of proteins and nucleic acids, adds challenges for the control of pharmaceutical preparations when using these molecules as ingredients in food supplements, cosmetics and in other fields. The chemical diversity of marine polysaccharides and habitat-related properties (salt tolerance, hyperthermostability, barophilicity and cold adaptivity) of marine biocatalysts acting on them are two topics of special importance from scientific and industrial points of view. Deep knowledge of both is necessary for exploitation of their bioprocesses.

Carbohydrate-active enzymes are families of structurally-related catalytic proteins and binding modules capable of degrading, modifying or creating glycosidic bonds. In the literature wide interest in these biocatalysts of marine origin appeared from results of research projects conducted since the 1960s, with particular focus on the selectivity of these enzymes in their hydrolytic reactions [1,2,3,4]. These marine enzymes often have represented a crucial tool in the hand of chemists struggling with the complexity of macromolecular structures before the era of high resolution NMR and MS facilities [5].

In different review articles carbohydrate hydrolyzing enzymes of marine origin still appear today as an important topic [5,6,7,8,9]. Their analysis enabled us to reach a main conclusion: these enzymes cover a broad range of individual application fields: (i) the biorefinery value-chain, where the provision of biomass is one of the most important aspects; (ii) the food industry to deal with the enzymatic procedures adopted in food manipulation; (iii) the selective and easy extraction/ modification of structurally complex marine molecules, where enzymatic treatments enable technology to improve efficiency and selectivity; and (iv) marine biomarkers. However, while searching the literature for the most recent of the above survey articles, dedicated to all enzyme classes [8], two particular aspects were noticed: (i) only half of the hits found were represented by original articles, being most of the remaining books or reference works and (ii) scientific interest in marine enzymatic processing was being often reported in non-specialized journals.

The importance of carbohydrate active hydrolytic enzymes in many application fields and the general limited presence of original articles in the literature search prompted us to undertake the refined updated analysis here reported focusing on carbohydrate active hydrolytic enzymes originating from marine environments. The rationale and limits of the literature search are detailed below.

## 2. Literature Search

The Science Direct database with access to 3800 scientific journals in major scientific disciplines was adopted for the analysis here reported. Moreover, science alert mailing lists were used, up to the manuscript submission, to update the analysis with the most recent results. The search for this review was conducted using the name of the enzyme spanning a two year (2016–2017) interval time and “marine” as limiting keyword. The W/in function (proximity operator, in = 15) was used as optimal value following a previously reported performance analysis [8]. The correct use of the proximity operator is an interesting point. Low-medium values are able to sort keywords (marine, name of the enzyme) in phrases while higher numbers are able to sort hits where keywords are present in long paragraphs, up to articles that could be not the target of the search. However, while in the previous analysis for general enzymatic processes [8] a value of W/50 was adopted, a value of W/15, with a minimal unrelated noise, was optimized here. Other less known polysaccharides do occur, but their related biocatalysts are either not yet found or not the object of studies and possibly did not fall within the literature-search terms for the given period.

## 3. General Analysis of Reference Material

**Figure 1:**
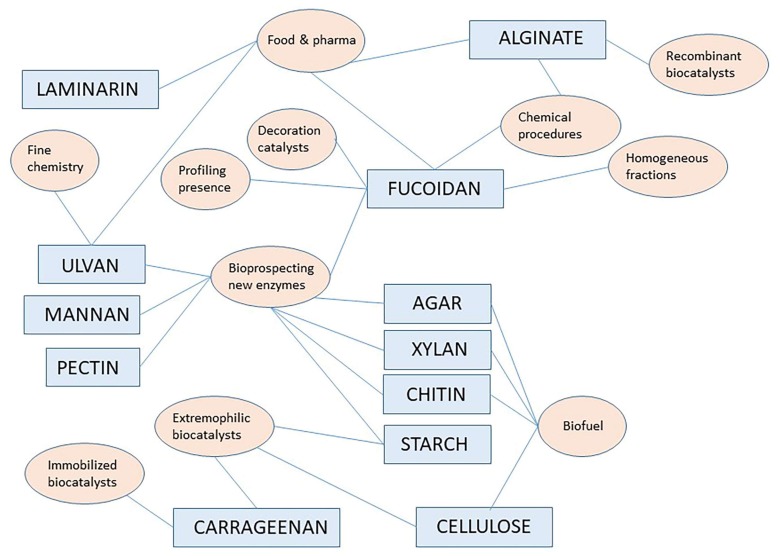
A pictorial style analysis of the most important aspects showing a network correlation based on polymers and research topics/fields indicated in keyword style as extrapolated from the two-years scientific material found for this review (see Section 2 for details on the literature search criteria).

In addition to W/in optimization, close inspection of abstracts and article text of resulting hits allowed assignment to either reference material or to original articles. General discussion on reference material (review articles, chapters in books and other reference works) is reported here picking up the most important contribution on leading topics. Original articles are discussed in different paragraphs below reported for specific polymer signaling, while there also other reviews in the starting parts to contextualize the whole topic. Figure 1 shows a pictorial style analysis of the most important aspects discussed in this review showing a network correlation based on polymers and research topics as keywords.

Commercial preparations of fucoidan with 95% degree of purity are available from a range of different species; however, modern aspects concerning sulfated polysaccharides were summarized in a recent review [10]. The advantages and importance of using enzymatic methods for modification of these molecules are highlighted. Hydrolases/glycosidases (e.g., fucoidanase, fucosidase, agarose, carrageenase, etc.), lyases, sulfotransferases and sulfatases can be used for this purpose. As for sulfatases, their concerted actions with carbohydrate-hydrolyzing enzymes can result in the production of neutral mono- and oligosaccharides used for energy consumption. Highlights on the diversity of sulfatases, revealed by genomic data mining, and a description of single marine polysaccharide sulfatases biochemically characterized to date are found in another interesting recent review [11]. As far as fucoidan structural complexity is considered, chemical procedures (e.g., hydrolysis, oversulfation and desulfation) for the production of “tailored” molecular species featuring specific biological activities have also been recently reviewed [12]. 

A chemical approach has also been reviewed for alginates; in the parent structure of these molecules new functional moieties can be inserted by utilizing the hydroxyl and carboxyl groups present per monosaccharide residue or aldehydes at the reducing chain end or possibly introduced by partial oxidation [13]. Marine carbohydrate active enzymes including lyases, sulfotransferases and sulfatases are necessary to really improve the value of sulfated polysaccharides from marine organisms [14]. A specific survey on polysaccharide lyases was also presented in the modern literature [15]. These important catalysts are produced by marine organisms belonging to various domains such as bacteria, fungi, and animals and have numerous biotechnological applications in fields such as food, paper industry, textiles, extraction, degumming of plant fibers, biomedicine, etc. They are studied for enzyme-assisted technologies in the production of bioactive extracts from green seaweed [16].

Chitinases represent a very interesting example, being chitin one of the underutilized resources in the world. Chito-oligosaccharides, including the monomer *N*-acetylglucosamine, are applied in various fields of chemistry, biomedical, biotechnology, agriculture, and environmental protection. The authors concluded that further research will focus on new biocatalysts, especially from extreme environments, and on suitable technologies for industrial production of these enzymes such as solid state fermentation [17].

The context of saccharification of red macroalgae is analyzed in two other recent reviews representing a comprehensive perspective for the efficient utilization of this biomass as a sustainable resource for production of bio-based products [18]. Enzymatic saccharification of solid agar waste produced in the agar industry by using two of the most widely used agarophytes, *Gracilaria verrucosa* and *Gelidium latifolium*, has also been studied [19]. Within this topic an effort to improve the secretion of a recombinant α-neoagarooligosaccharide hydrolase (AgaNash), from *Cellvibrio* sp. OA-2007, expressed into the YPH499 strain of *S. cerevisiae* is of interest. Even using a non-marine derived hydrolase, it was found possible to hydrolyze neoagarobiose with a remarkable efficiency of 84%, producing galactose, a directly fermentable sugar for yeast. Ethanol was produced from neoagarobiose at concentrations of up to 1.8 g/L in the direct process [20]. 

An overview of the great potential of microalgae to produce industrial enzymes has been also recently compiled [21]. Among future perspectives, a growing number of marine carbohydrate active enzymes can be discovered from these organisms although a large part of their genetic resources remains unexplored. It is concluded that microalgae biomanufacturing will be an important approach to scale-up industrial production of biocatalysts. 

Cell wall polysaccharides from green algae belonging to species of *Ulva* represent an important amount (38–54%) of which about 8–29% constituted by ulvan. Different repeating sequences based on disaccharidic units of rhamnose, glucuronic acid, iduronic acid, xylose, and sulfate in variable proportions are found in different samples from several Ulvales. Not minimizing NMR spectroscopy methodology, the chemo-enzymatic degradation approach has been widely used in these structural assessments to conclude that ulvans are a family of chemically related branched molecules of broad distribution in term of charge density and molecular weight. Ion-binding and gelling properties of these macromolecules, strictly related to structural features, have a large impact on applications in the food/feed, pharmaceutical, chemical, aquaculture, and agriculture fields. Furthermore, their composition based on rare sugars makes them useful precursors for stereochemically defined building blocks in the fine chemistry domain. All these aspects have been recently reviewed in a complete survey analysis [22]. 

In the most modern report on amylases of microbial origin [23] different mentions of the importance of marine derived examples are included describing molecular methods (metagenomics) for discovering novel enzymes. Indeed, a specific report is also present within the important modern reference material. The marine ecosystem is confirmed as a huge natural reservoir for novel and useful amylases. Some with specific characteristics of interest in industry (cold activity, salt tolerance, pH and temperature stability) have been identified from marine bacteria and different species of marine fungi and yeasts. Amylases were recognized also in marine animals in all fish species and their activity being related to feeding habits [24].

### 3.1. Agar

The structure of the agar can be briefly described as formed by a mixture of two polysaccharides named agarose and agaropectin. The first is composed of repetitive units of β-d-galactose (B-GAL) and 3,6-anhydro-α-l-galactose (3,6-AG). B-GAL is connected by a β-1,4 glycosidic linkage and 3,6-AG is linked to the next B-GAL by α-1,3 linkage. Few variations are present, including a low content of sulfate groups. Agaropectin is similar in structure with a lower molecular mass below 20,000 Daltons. Some hydroxyl groups of 3,6-AG moieties are sulfated, methoxylated or possess pyruvate residues. Agarose and agaropectin are present in variable proportions in agar, depending on the origin of the raw material.

Agar has a wide variety of uses. Due to its stabilizing and gelling properties has been mainly used in microbiological media. In Japan has been used as a food for several hundred years. It is a Generally Recognized As Safe (GRAS) food additive, used in pastry and cheese manufacturing, etc. Oligosaccharides originating from agar exhibit antioxidative and other biological activities.

Agarases are important catalytic species classified as α- and β-agarases according to the glycosidic linkage broken, producing two different moiety types. The former hydrolyze agarose at the α-1,3 linkages and produce agaro-oligosaccharides that have 3,6-anhydro-l-galactose residues at their reducing ends. The other type hydrolyze agarose at the β-1,4 linkages and produce neoagaro-oligosaccharides with d-galactose residues at the reducing ends. β-Porphyranases are more specific, hydrolyzing the β-1,4 bonds in porphyran where the backbone consists largely of β-GAL followed by α-l-galactopyranose-6-sulfate (G-L6S). 

The general machinery of bond cleavage performed by agarases is increasingly well studied. However, a less clear point is represented by the mode of enzymatic action on insoluble substrates. Among different important applications of these enzymes, recovery of DNA from agarose gel, production of derived oligosaccharides (broadening possible applications), degradation of cell walls (for preparation of protoplasts), and improvement of modern extractive processes, can be listed [25].

Members of the *Agarivorans* genus are classical examples of agarose producers. A new strain of *Agarivorans gilvus* possesses three interesting agarase activities, and its complete genome sequence has been published [26]. Among new examples found in the two year literature span, others should be cited: *Acinetobacter junii* PS12B from which an extracellular agarase was identified [27], the alkaliphilic bacterium *Cellvibrio* sp. WU-0601 with a neoagaro-oligosaccharide-specific hydrolase (EC 3.2.1.159, α-NAOS hydrolase) that hydrolyzes short oligosaccharides (from neoagarobiose to neoagarohexaose) at the α-1,3 linkage [28], *Cellulophaga lytica* DAU203 with three β-agarases [29], *Cellulophaga omnivescoria* [30], *Flavobacteria* and *Gammaproteobacteria* libraries, where different agarases and other algal-associated enzymes were identified [31], an alkaline β-agarase from the marine bacterium *Stenotrophomonas* sp. that degrades agarose into neoagarobiose, neoagarotetraose and neoagarohexaose as predominant products [32] and an agarase of the GH family 16 from the marine bacterium *Aquimarina agarilytica* ZC1 [33] producing neoagaro-tetraose, neoagaro-hexaose and neoagaro-octaose as main hydrolysis products.

Different articles found have a more practically oriented nature, reporting applications of already known activities. A combined acid and enzymatic hydrolysis using a recombinant agarase to produce total reducing sugars from *Gracilaria verrucosa* was published [34]. A 5% increase (47.4%) with respect to the previous value of reducing sugars obtained by commercial enzyme is claimed for this biorefinery-oriented application. A similar application appeared for the same enzyme acting on *Gracilaria lemaneiformis* is also reported [35].

In our results biochemically-oriented original studies were also found testifying to the importance of basic research to improve our knowledge of this class of enzymes. Flammeovirga is a recently defined bacterial genus possessing a potent ability to degrade marine complex polysaccharides. AgaB, was successfully cloned from *Flammeovirga* sp. SJP92 and expressed in *Escherichia coli*. This β-agarase has a high pH stability under neutral to mildly alkaline conditions (pH 7.0–10.0) with tolerance to higher concentration of denaturants compared to that usually reported for agarases. Eventually both characteristics are of importance for industrial applications [36]. As for thermostability, a study investigating the solvent accessibility for β-agarase has been published. Establishment of location of residues and solvent accessibility is judged to play a crucial role in engineering thermostability in the so-called ‘semi-rational’ method that combines computational techniques to traditional enzyme engineering methods to produce resistant biocatalysts [37].

Another interesting study is one regarding the enzyme endo-β-agarase I from *Microbulbifer thermotolerans* JAMB-A94, previously expressed as a catalytic domain (GH16) without a carbohydrate-binding module (CBM). A new expression fusing the catalytic protein to its own CBM6 or to another carbohydrate binding module (CBM13 from *Catenovulum agarivorans* YM01) enhancing the catalytic efficiency, is reported. The potential in the biofuel value chain was investigated using this enhanced catalyst in pre-hydrolyses of both agar and agarose during the first step of saccharification [38].

A study investigating the biological functions of three neoagaro-oligosaccharides, namely neoagaro-biose, neoagaro-tetraose, and neoagaro-hexaose prepared by hydrolyzing agar with two recombinant β-agarases, DagA and DagB from *Streptomyces coelicolor* A3, was also published. No antiscavenging activity against DPPH and a very weak antibacterial activity were observed for the hydrolyzed products [39].

### 3.2. Chitin

Chitin is a polysaccharide formed by β-(1,4) linked *N*-acetylglucosamine units. It represents the second most abundant polysaccharide found in Nature after cellulose. Three polymorphic crystalline structures of chitin exist—α, β and γ—according to the ways the molecular chains are organized in the crystal cell. Chitin is the major component of crustacean and insect skeletons and fungal cell walls. In the marine environment chitin is particularly important, representing a nutrient source for the ecosystem. Marine wastes are considered a great source of chitin; in fact some 80,000 tons of chitin are produced from marine wastes every year. Production of high value-added compounds from this material has a vital connection to chitinases. More recently also total bioconversion of chitinous material to ethanol has focused interest in the energy industry.

The biotechnological applications of chitinases include isolation of protoplasts from fungi and yeasts by cell-wall degradation; access to *N*-acetyl-d-glucosamine and other pharmaceutically important derived chitooligosaccharides via sustainable alternatives to chemical hydrolysis of the polymer; bioprocessing of marine chitinous wastes; and other more specific uses such as biocontrol of fungal phytopathogens or harmful insects. Chitinases break protective coats weakening the defense systems of several pathogenic microorganisms and insects. Some oligosaccharides derived from chitin with a specific degree of polymerization possess antitumor, hypolipidemic, haemostatic and antimicrobial activities [17].

Chitinases have been isolated from bacteria, fungi, plants, insects and animals and are classified into two glycoside hydrolase families, GH18 and GH19 [40,41]. Most members are mesophilic proteins not possessing the features required to face harsh industrial conditions. Therefore, novel chitinases are seen of great importance from biotechnological point of view in that they may possess unique characteristics, such as optimal acidic pH and operative thermal stability. A recent interesting review article on fish chitinases has been published dealing with the enzyme(s) contained in the stomach and with physiological degrading function of chitin from diet. Authors suggest that some enzymes could serve multiple roles (i.e., defense against pathogens) not strictly linked to digestion [42]. This is an important aspect with economic impact, as discussed in a recent report on the complete genome sequence of a fish pathogen. The investigated genomic information of *Aeromonas veronii* TH0426 could reveal not only the pathogenic mechanism associated with yellow catfish, but could serve to avoid economic losses in fish farming and give insights on potential application in producing chitinase and other enzymes [43]. On the same line of research other genes for chitinases have been characterized in turbot [44]. Related chitinases could serve to increase turbot’s resistance to diseases.

A novel chitinase has been recently purified from the marine bacterium *Paenicibacillus barengoltzii* CAU904. It hydrolyzed colloidal chitin to yield chitobiose to chitotetraose at the initial hydrolysis stages indicating that it is an endo-type chitinase. At the end of the reaction, chitobiose and *N*-acetyl-d-glucosamine were mainly produced. The biocatalyst was highly active at pH 3.5 and stable in a wide range of pH values (3.0–9.0). The optimal temperature was 60 °C [45]. From the same organism, another enzyme of the same type allowed to produce 21.6 mg mL^−1^ of chitobiose at the highest conversion yield of 89.5% (*w*/*w*) making it a good candidate for chitobiose production [46]. Other marine chitinases were signaled from a marine *Bacillus* sp. originating from the Red Sea [47,48]. Here an interesting tolerance to some organic solvents such as ethanol, acetone, and isopropanol was reported(the enzyme retained up to 70% of its original activity). However, nearly full inhibition by other solvents such as chloroform, diethyl ether, ethyl acetate or diethylene dioxide was detected. Substrate properties of the enzyme were also studied [49] indicating high affinity toward squid chitin with respect to colloidal chitin. 

A marine fungal chitinase from *Aspergillus terreus* isolated from a marine sediment in Saudi Arabia was recently described. The purified protein showed good pH (retained 80% of its activity in a pH range from 5.0 to 8.0) and thermal stability (retained 42% of its activity when treated at 70 °C for 60 min). An interesting wide spectrum of antimicrobial activities of this purified chitinase was detected. The authors indicated that the enzyme can be evaluated as a biocontrol agent against some pathogenic microbes other than in the commercial preparation of *N*-acetyl-d-glucosamine [50].

An interesting report on chitin binding domain of the chitinase from *Vibrio harveyi*, a marine bacterium producing high levels of chitinolytic enzymes recently appeared. The finding sheds some light on the way the chitin binding domain acts. The gene fragment encoding it was cloned and the analysis showed correct folding for functional characterization. β-Colloidal chitin was found to have the greatest affinity with respect to α-chitin, however chitosan (with free amino groups) was barely bound. Authors concluded that the *N*-acetamido groups of the native form should play a role in chitin-chitinase interactions [51].

From the soil of a shellfish industry plant, a chitinase producer was recently individuated (*Bacillus pumilus* RST25). Extracts and partially purified chitinase exhibited antagonism against the plant pathogen *Fusarium solani* and *Aspergillus niger* and the enzyme suppressed the fungal infections of *Triticum aestivum,* thus revealing the interest for the biocatalyst as a possible biocontrol agent against fungal phytopathogens [52].

It has also been recently reported a study demonstrating the potential production of *N*-acetyl-glucosamine from α-chitin using chitinases from ten marine-derived *Aeromonas* isolates. Yields of up to 93% were obtained using crude chitinase extracts from *Aeromonas caviae* CH129 at 37 °C, pH 5.0, 2% (*w*/*v*) colloidal chitin [53].

### 3.3. Starch

Starch is a common polysaccharide and its structural details can be found in the general textbook literature. Amylases are the biocatalysts for the hydrolysis of starch; α-amylase (EC 3.2.1.1), β-amylase (EC 3.2.1.2), glucoamylase (E.C 3.2.1.3), isoamylase (EC 3.2.1.68) and glucosidases (EC 3.2.1.20) being the best known types. Great interest in these enzymes, even in the form of an enzymatic cocktail, is found for biological pretreatments of biomasses in biorefinery pipelines; entire microbial communities associated with marine organisms are also studied in this respect. There is a wide range of known applications for amylases that can be easily found in common applied biotechnology book, including the textile industry, treatment of cellulosic materials, leather, and detergents, just to mention few of them. However the pharmaceutical and chemical industries are also interested. Amylases have been recognized in marine animals, in all fish species and their activity is related to feeding behaviour. Novel starch-acting enzymes are of interest, in particular those from different marine sources for their important industrially oriented properties [54]. Cold activity, salt tolerance, pH and temperature stability of specific amylases from marine bacteria and from different species of marine fungi and yeasts are interesting characteristics to this aim.

Production of pullulan at 36 g/L was reported by using potato starch and a marine cold-adapted amylase from *Aureobasidium pullulans*. Pullulan itself, including derivatives, is an important molecule in the food, cosmetic, and pharmaceutical industries (biomedical applications) due to structural flexibility of the interconnected α-(1-6) maltotriose units, reflecting in interesting properties (production of thin transparent films, oil resistance and impermeability to oxygen) [55].

A recently identified amylase from the marine bacterium *Catenovolum* sp. X3 is cold-active and possesses alkali and solvent-tolerance, a feature that could enhance the production of biofuels from starch. The enzyme has been expressed in *E. coli*, characterized and found to facilitate starch hydrolysis to maltose and oligosaccharides, in turn applied to produce biohydrogen [56]. Similar characteristics were found in another novel alkalophilic α-amylase from a deep-sea originating bacterium, *Luteimonas abyssi*. The biocatalyst is highly active compared with other amylases, especially at low temperature (8881 U/mg, a higher activity than other alkalophilic amylases) and is able to retain its activity without calcium, a favorable feature in the detergent industry where softened waters are in use [57]. Similarly, from a hot spring located in Odisha, India, the microorganism *Exiguobacterium* sp. SSB11, produced a thermostable α-amylase that has been purifed and characterized discovering an interesting Ca^2+^ independent activity. It is considered the first example of an *Exiguobacterium* sp., producing alkaline amylase at an alkaline growth pH. Indeed this bacterium has been proposed as a model organism in the study of molecular basis of alkalophilicity. The ability of this biocatalyst to resist to high temperature and broad range of pH, suggests its biotechnological application in different industries [58]. 

From the fish *Siganus canaliculatus*, an herbivorous marine teleost, an α-amylase gene was cloned and its tissue expression studied for the first time [59]. This interest is in the frame of a study aimed to refine knowledge of α-amylase function in herbivorous marine fish for the development of low-cost and effective feeds incorporating seaweed as a dietary ingredient.

The production of α-amylase in marine protists, thraustochytrids, has been studied using response surface methodology (RSM) analyzing the most influencing process variables on possible cumulative interactive effect of nutritional components (glucose, corn starch and yeast extract) [60].

### 3.4. Laminarin

Discussions related to laminarin are grouped with a general search on glucan. Results for marine glucanases are still limited to the two years interval time as for other enzymes. However, since very few products were found for laminarinases, the search interval time was extended back to 2014, while enzymes acting on cellulose are discussed in a separate paragraph below.

Laminarin is a water-soluble polysaccharide constituted by 20–30 glucopyranose units joined by β-1,3-linkages with about 5% of β-1,6-linkages. Individual chains have an average of 1.3 branches per molecule. Other glucans with this general structure exist, such as curdlan that is a high MW water-insoluble 1,3-β-glucan containing <1% 1,6-β-linkages. 1,3-β-Glucans are of increasing interest because of their important biological functions as antitumor agents and immune response modulators. Among their commercial applications, anti-viral activity useful in agricultural field, actions as dietary fiber and substrate for prebiotic bacteria, etc., can be listed. Sulfation of hydroxyl groups seems to increase the anticoagulant action of these molecules. Particular studies on the antioxidative potential of laminarin in meat products are also found in the literature [61].

β-1,3-Glucanases represent a well-known class of enzymes widely spread in bacteria, fungi, plants and marine animals. These are hydrolases specific for *O*-glycoside bonds between the 1,3-linked glucopyranose residues found in homoglucans (laminarin, curdlan) and mixed-linked β-glucans. The functions of these enzymes are diverse and quite distinctive in different producers according to their kingdom. In pathogenic bacteria, these enzymes participate to the cell wall digestion process. In higher plants, they cleave glucans in seeds and also act as inducible defense enzymes. They are involved in autocatalysis of extracellular matrix glucans in fungi and in yeast cell development. In the animal kingdom such enzymes are commonly found in marine echinoderms where take part in the digestion of algae-based food and also play some important roles in embryogenesis. β-1,3-Glucanases in several marine bacteria and fungi have been cloned and sequenced. Some representatives are also commercially available and adopted in extractive process technology of bioactive extracts from macroalgae [62].

Recently a practical application of oligosaccharides from *Laminaria japonica* has been reported. These molecules were added to pullulan coating and the material used to preserve cherry tomatoes. The authors found that inserted oligosaccharides have a dose-dependent beneficial effect increasing shelf life of cherry tomatoes by inhibiting respiration, retarding tissue softening and weight loss [63]. In another study is reported a potential application of a laminarinase from the marine bacterium, *Saccharophagus degradans* in direct ethanol production from laminarin by a co-culture system composed of two yeast strains displaying marine laminarinase and β-glucosidase activity [64,65]. Glucanases from marine animals are of great interest. They have been thoroughly investigated in *Aplysia* sp. in the past [66]. An interesting laminaribiose-hydrolyzing enzyme from the digestive fluid of the marine gastropod *Aplysia kurodai* was identified with impact into the process of complete degradation of laminarin to simple monosaccharides. The biocatalyst rapidly hydrolyzed laminaribiose while acting very slowly on cellobiose, gentiobiose and lactose, and was completely inactive on sucrose and maltose. Interestingly, with his high transglycosylation activity it can produce a series of laminari-oligosaccharides larger than laminaritetraose from laminaribiose (a donor substrate) and laminaritriose (an acceptor substrate) [67].

Another interesting report is one about characterization of Gly5M, a novel β-1,3-1,6-endoglucanase from the marine bacterium *Saccharophagus degradans* 2–40 (T). The enzyme belongs to family 5 (GH5) and possesses β-1,3-, β-1,6-endoglucanase activities being capable of cleaving glycosidic linkages of laminarin and pustulan (a β-1,6-glucan derived from fungal cell walls) as substrates. This enzyme also showed transglycosylation activity toward β-1,3-oligosaccharides when laminarioligosaccharides were used as substrates. Since laminarin is the major form of glucan storage in brown macroalgae, Gly5M could be used to produce glucose and laminarioligosaccharides, using brown macroalgae for industrial purposes [68].

### 3.5. Alginate

Alginates are made of two 1,4-β-linked pyranose ring units—β-d-mannuronic acid (M) and α-l-guluronic acid (G)—which are C-5 epimers each other. They are arranged in poly-G and poly-M blocks of various length, often occurring as random sequences, interspaced with regions of alternating sequences (MG block). Both the proportion and arrangement of blocks are dependent on the source and have a direct impact on the physical properties of the polymer; in particular gel properties are largely influenced by the content and length of the G blocks. Ionic cross-linking with divalent cations is responsible for the formation of hydrogels. 

Alginates are produced in brown algae (Pheaophyceae, such as *Laminaria hyperborea*, *Laminaria digitata*, *Laminaria japonica*, *Ascophyllum nodosum*, and *Macrocystis pyrifera*) as structural polysaccharides. In certain genera of Gram-negative bacteria (*Azotobacter* and *Pseudomonas* sp.) exocellular alginates have different protective functions, and they could act as virulence factors. However, purified alginates are relatively inert toward cells and biomolecules, providing good biocompatibility. Two hundred different types are manufactured with numerous current applications; hydrogels of alginates bear resemblance to extracellular matrices of living tissues, they can maintain a physiologically moist microenvironment and minimize bacterial infection at the wound site, thus facilitating wound healing. Hydrogels can also be used for delivery of bioactive agents, immuno-isolation of cell transplants, in vitro tissue engineering, 3D-bioprinting, etc. [69].

Alginates are degraded by a group of enzymes, alginate lyases, that catalyze the β-elimination of the 4-*O*-glycosidic bond forming a double bond between C-4 and C-5, resulting in unsaturated uronic acid-containing oligosaccharides. Short oligomers possess interesting bioactivities. Endotype and exotype alginate lyases are produced by alginate-degrading bacteria that are also able to depolymerize alginate into its constitutive monosaccharides in a complete manner. Exotype in particular are key enzymes for total utilization of alginate in saccharification. They act on oligoalginates produced by the endotype counterparts producing monomers for ethanol production in biofuel applications.

In a comparative study Hirayama et al. settled the question of the characterization of several exotype alginate lyases studied independently (i.e., their activities have not been assayed under the same conditions or using the same unit definition) rendering it difficult to correlate enzymatic actions. Interestingly they found halotolerance for Alg17c derived from the marine bacterium *S. degradans*. Halotolerance is obviously a preferred property of an enzyme under industrial point of view for applications in the utilization of brown macroalgae as a carbon source since algal materials are likely to contain high salt concentration [70].

In 2016 Zhu et al. reported the characterization of an extracellular alginate lyase from marine *Microbulbifer* sp. ALW1. Disaccharides and trisaccharides produced from the polymer indicated that it could be a good tool for preparation of alginate oligosaccharides with low degree of polymerization [71]. The authors measured the antioxidant activity of hydrolysates produced by this enzyme.

The very active research group of Ojima recently reported a complete study on the overall alginate degradation metabolism in *Flavobacterium* sp. UMI-01. Degradation process from alginate to unsaturated monosaccharides was studied in detail. From their previous study, FlAlyA was purified and characterized as an endolytic alginate lyase. Crude extracts of *Flavobacterium* sp. UMI-01 were found to be capable of producing 4-deoxy-l-erythro-5-hexoseulose uronic acid from short oligosaccharides. In the mentioned report they described four alginate lyases, namely FlAlyA, FlAlyB, FlAlyC, and FlAlex, found in the genomic sequence, and characterized them as recombinant proteins. These enzymes have distinct roles for complete degradation of alginate to 4-deoxy-l-erythro-5-hexoseulose uronic acid. Degradation of polymer started with FlAlyA action attacking the alginate in an endolytic manner, producing oligosaccharides. 4-deoxy-l-erythro-5-hexoseulose uronic acid was in turn generated as a final degradation product through the function of three enzymes FlAlyB, FlAlyC, and FlAlex, which exhibited specific substrate preferences; FlAlyB acted preferentially on polyM- while FlAlyC, and FlAlex are polyMG-, and polyG-specific enzymes, respectively [72].

In alginate biosynthesis GDP-mannuronate can be considered a key substrate. It is polymerized to polymannuronate and this polymer is subjected to mannuronan C5-epimerase (MC5E) to transform parts of M- to G-. The same group of Ojima recently published the first report on the characterization of a MC5E activity present in eukaryotes, being structure and function of these enzymes well characterized only in bacteria. Activity of the enzyme on M-block was simply analyzed by ^1^H NMR spectroscopy; anomeric protons of G residues were clearly differentiated by this technique. Interestingly the results using a specific antibody revealed that multiple MC5Es are expressed in brown alga *S. japonica*, and the expression patterns is dependent on the portions of algae [73].

In aquaculture of sea cucumber, used as an important fishery resource in Far East countries, *Laminaria japonica* is used as an animal feedstuff. With the aim of reducing the alginate content of *Laminaria*, not easily digested by echinoderms, *Bacillus amyloliquefaciens* WB1 was isolated from marine mud by virtue of its ability to utilize sodium alginate as the sole carbon source. A study based on surface methodology optimization recently appeared investigating eight factors affecting growth and alginate-degrading capacity of this microorganism thus obtaining optimal parameters for fermentation time, beef extract content, etc. Results indicate ca. 60% of alginate degradation [74].

Five different recombinant alginate lyases have been evaluated for their effect on enzymatic liberation of glucose from the brown macroalgae *Macrocystis pyrifera* from Chile and *Saccharina latissima* from Norway. These enzymes belonging to polysaccharide lyases families PL7 and PL18, were previously expressed in *E. coli*. Even though for saccharification of pretreated algae only cellulases are needed to achieve high glucose yields, these experiments showed that the use of recombinant alginate lyases in combination with cellulases increased the release of glucose from untreated seaweed [75].

In aquaculture field interest for the growth of abalone *H. rubra* recently increased. One of the big issue is the growth rate to reach marketable size of abalone, related to the amount of digestive enzymes present in the organism for digestion of diet. Supplementation with digestive enzyme producing bacteria has been one of the approach used in this field. A study aimed at the screening and isolation of alginate lyase producing bacteria from the gastrointestinal tracts of hybrid abalone recently appeared. Authors selected a probiotic strain with high alginate lyase activity. The ingested probiont identified as *Enterobacter ludwigii* was able to pass through the intestinal tract where it was able to contribute to breaking down food particles. The study shows its high viability even in manufactured pellets stored at 4 °C for 1 week [76].

### 3.6. Fucoidan

Fucoidans are a family of sulfated homo- and heteropolysaccharides found in various species of brown algae and exhibiting important biological functions. They are mainly composed of sulfated l-fucose with less than 10% of other monosaccharides. Their complex structures differentiated in the degree of branching, substituents, sulfation and type of linkages with fine structural details that are depending on the source and geographical origin; apart fucose and sulfate, small proportions of uronic acids, galactose, xylose, arabinose and mannose can be present. Two main basic structures are common: repeated (1-3)-linked α-l-fucopyranosyl residues or alternating (1-3)- and (1-4)-linked α-l-fucopyranosyl residues. Fucoidans are considered cell wall reinforcing molecules with some role in protection against desiccation. *Fucus vesiculosus* brown algae contains the highest concentration of fucoidans (up to 20% on a dry weight basis). Estimates report that 40% of dry weight can be represented by these polymers for isolated algal cell walls. Fucoidan hydrolases are important enzymes for structural identification of complex structures of fucoidan.

Fucoidans are known to possess numerous biological properties with many potential applications for human health, as these molecules show anticoagulant, anti-viral and anticancer properties. Activities are not only related to molecular weight and sulfated ester content (role of the charge of the molecule) but also to position of sulfate groups and content of glucuronic acid. For this very aspect sulfatases are other important enzymes in this context [11]; however the search for these activities resulted in no original articles for the two year time span considered.

In a recent technology report related to this topic, a microarray-based technology was used for profiling a selection of marine animals using antibodies raised against fucoidan. This is important in the context of studies aimed to reveal the biological importance and pharmacological potential of these glycans from marine organisms; in fact, there are many unanswered questions regarding distribution, function, and evolution of these molecules [77]. Other sulfated polysaccharides are present in marine organisms such as carrageenans in red algae and ulvans in green algae. The microarray-based glycan profiling showed that fucoidan epitopes typical for brown algae are relatively widely, but not ubiquitously present in diverse marine organisms. The presence of two fucoidan epitopes in Porifera, Mollusca, and Chordata and in particular the spatial distribution of these epitopes in the sponge *Cliona celata* were reported.

Among other original articles to be discussed here one of the most recent is from Silchenko et al. [78] in which authors reports on structures and bioactivity (anti-cancer) of five fuco-oligosaccharides from *Sargassum horneri* that were prepared by fucoidanase activity from the marine bacteria *Formosa algae*, expressed in *E. coli*. While interesting details on polymer structure were obtained by the mild enzymatic methodology adopted, authors concluded that fragments obtained with the degree of polymerization of 4 to 10 are unable to interact with the target tumor cells effectively.

A study examined the inhibitory capability of 11 different fucoidans (*Sargassum thumbergii*, *S. honeri*, *S. ringgoldianum*, *S. siliquastrum*, *S. graminifolium*, *S. kjellmanianum*, *Fucus vesiculosus*, *Ascophyllum nodosum*, *Lessonia nigrescence*, *Kjellmaniella crassifolia*, and *Costaria costata*) on α-glucosidase and α-amylase. The context is the potential application of fucoidan for the treatment of diabetes or obesity and the interest about structure-activity relationships in experiments on animal models. Fucoidan from *Fucus vesiculosus* showed the highest α-glucosidase inhibitory activity, with an IC_50_ value of 67.9 μg/mL [79].

A novel α-l-fucosidase from a marine bacteria *Wenyingzhuangia fucanilytica* has been recently identified. The authors, after analyzing the importance of the study of α-l-fucosidases for manipulation of fucose-containing glycoconjugates and polysaccharides such as fucosylated chondroitin sulfate, human milk oligosaccharides, erythrocyte surface antigens, etc., indicated that this enzyme was active in the hydrolysis of α-fucoside linkage of the synthetic substrate pNP-fucose and in others molecules such as Gal-β-1-3-(Fuc-α-1-4)-GlcNAc and in partially degraded fragments of fucoidan [80].

A similar enzyme, a lysosomal α-l-fucosidase from the fresh water mussel *Lamellidens corrianus*, has also been reported. The purified enzyme exhibits considerable thermal stability even though authors did not report any actions on different substrates other than synthetic pNP-fucose [81].

To demonstrate interest for this topic of fucoidan degradation the first report of an enzyme capable of catalyzing the deacetylation of fucoidan should be mentioned even though the enzyme, strictly speaking, is not a carbohydrate active enzyme. The enzyme is found in the cell-free extracts of strain *Luteolibacter algae* H18 and was produced in *E. coli*; it catalyzes fucoidan deacetylation, but not desulfation and degradation into lower forms. In addition to fucoidan deacetylation, the enzyme catalyzed the hydrolysis of pNP esters with organic acids with pNP acetate as the best substrate [82].

### 3.7. Carrageenan

Carrageenans are sulfated polysaccharides obtained by extraction of certain *Rhodophyceae* (red seaweed) using water or alkaline water. They consist mainly of sulfate esters salts of galactose and 3,6-anhydrogalactose copolymers. The disaccharide sequence is composed of a 1,3-linked 3,6-anhydro form of α-d-galactopyranose (A residue), and 1,4-linked β-d-galactopyranose residues (B residues). Carrageenans are distinguished from agars with similar structure in that their anhydrogalactoses of A units are in the D-form whereas they are in the L-form in agars. There are three general structural forms of carrageenans (κ, λ and ι) that possess their own gelling property due to sulfate groups; one is present in κ-carrageenan, two in ι-carrageenan and three in λ-carrageenan. However, intermediate structural hybrids are present in fact there are 15 Greek letters used to describe different carrabiose moieties; moreover 4-sulfate or unsulfated and 2,6-disulfate of B residues and 3,6-anhydro-2-sulfate are also possible.

Carrageenans have been widely used in food and other industries as thickening and stabilizing agents for over six centuries. Originating long ago, a large number of medical applications are also described for these polymers; they are used as suspension agents and stabilizers. Many reports exist about their anti-coagulant activity and inhibiting platelet aggregation action.

Enzymes that degrade carrageenans, namely κ, ι and λ-carrageenases, have been isolated from various marine bacteria. They are endohydrolases that cleave the internal β-(1-4) linkages of carrageenans yielding products of the neocarrabiose series.

Among recent reports, one reporting the psychrophilic marine bacterium *Flavobacterium* sp. YS-80–122 as a producer of a ι-carrageenase was found. High activity of the recombinant form of this enzyme has been reported. It is an endo-type ι-carrageenase that hydrolyzes β-1,4-linkages of ι-carrageenan with high specificity, yielding neo-ι-carratetraose as the main product in high yield, better than previously reported values. This catalyst has been proposed in industrial production of oligosaccharides of this type. Among other interesting characteristics such as cold-activity and thermo-tolerance, the action of this catalyst even in absence of NaCl is worth noting, even though the activity is enhanced in the presence of sodium chloride [83].

Alga-associated cultivable microbiota has been studied to better assess the underexplored potential of associate microorganisms possessing interesting catalytic activities, not only related to carbohydrate active enzymes. A plurigenomic approach using the brown alga *Ascophyllum nodosum* enabled to reveal many interesting biocatalysts acting on carrageenans. Two ι-carrageenases represent in fact new members of a poorly known GH82 family [31].

A study was developed regarding a high-efficiency method to prepare carrageenan oligosaccharides from *Eucheuma cottonii* using cellulase and a recombinant κ-carrageenase. The context of this paper is the attention to carrageenan oligosaccharides for their potential to inhibit viruses, tumors, and coagulation. Authors started with assumption that the preparation of these oligosaccharides would be significantly simplified if dried seaweed could be used directly as a raw material; in this paper they developed a multienzymatic approach by using a consecutive two-steps enzymatic hydrolysis, first with cellulase and then with a recombinant carrageenase. A positive aspect is represented by their methodology causing little structural damage to carrageenan oligosaccharides because harsh chemical treatment is not required [84]. 

An interesting report has been recently published on the immobilization of these enzymatic activities. κ-Carrageenase was immobilized onto magnetic iron oxide nanoparticles. These immobilization nano-agents are receiving attention because of their large surface area, easy separation from reaction solution by magnetic field and possibility of chemical functionalization of surface. The enzyme used is κ-carrageenase produced by *Pseudoalteromonas carrageenovora* CICC 23819. Immobilization process increased thermal, pH, and storage stabilities of immobilized catalyst at some extent although the κ-carrageenase exhibited lower affinity [85].

### 3.8. Xylan

Xylans are polymers constituted by a backbone of xylose units. The most common β-1,4 linked xylans are present as basic hemicellulose component of plant cell walls and represent one-third of all renewable organic carbon on earth. β-1,3-Linked xylans also exist, but are less common. Mix-linked (1 → 3)/(1 → 4) xylans are present in the edible red alga *Palmaria palmata*. 

Complete degradation of β-1,4 linked xylans requires the synergistic action of a variety of glucoside hydrolases, including xylanases, β-xylosidases, α-glucuronidases, α-arabinofuranosidases for the different glycosidic linkages present and acetylxylan esterases to get rid of decorating groups of the basic structure. Xylanases are involved in providing sources of carbon and energy for the organisms possessing these enzymes and are involved in the growth, maturation, and ripening of cereals and fruits. They are widely used in different industrial fields (bioconversion of lignocellulosic materials into fermentable products, clarification of fruit juices and wines, papermaking, fuel production, quality improvement of dough and bread). In recent years, salt-tolerant xylanases have become an interesting research topic because they can potentially be used in salt-rich environments, such as the processing of marine foods and in marine wastewater treatment [86]. 

Enzymes acting on 1,3-β-xylan were found in different marine microorganisms of the genera *Vibrio* and *Alcaligenes*. β-(1-3)- [87] and β-(1-4)-linked [88] xylooligosaccharides are also therapeutically important compounds.

*Microcella alkaliphila* JAM-AC0309 is a new isolated xylan-degrading bacterium from a deep subseafloor core sediment in Japan; the whole genome was sequenced containing 16 genes encoding for enzymes in xylan utilization: namely two xylose-responsive transcription regulator genes, three β-1,4 xylanase genes, one α-l-arabinofuranosidase gene, two α-glucuronidase genes, two sugar ABC transporter genes, three xylose isomerase genes, and three xylulose kinase genes. Authors emphasized the interest for this strain in industrial processing [89]. It retained more than 77.4% of activity in the presence of 0.25–4 M NaCl displaying more than 47.2% relative activity after incubation in the presence of 5 M NaCl at 37 °C for 10 min.

From an Antarctic marine bacterium, *Psychrobacter* sp. strain 2–17 a new cold-active xylanase was cloned, recombinantly expressed in *E. coli* strain BL21(DE3) and characterized. Mutants were also produced after molecular dynamic simulation of flexible regions of the protein to successfully influence thermolability of the enzyme [90].

From a sediment of mangrove environment an actinobacterium *Streptomyces olivaceus* was recently isolated; optimization of xylanase production, according to culture conditions, and xylanolytic properties were reported. The purified enzyme was used in xylanolysis of agro-wastes (sugarcane juice) to reducing sugar for bioethanol production [91].

*Geobacillus* spp. are Gram-positive thermophilic bacteria of great interest for their potential in biotechnological applications as sources of thermostable enzymes. They can be found in various terrestrial and marine environments. A β-xylosidase, GbtXyl43B, from *Geobacillus thermoleovorans* expressed in *E. coli* was used for the hydrolysis of alkaline extracts of corncob. The enzyme is known to act on soluble and insoluble xylans producing free xylose, xylo-oligosaccharides and arabinose [92].

A statistical approach has been used to optimize the production of a xylanase activity by the marine *Streptomyces* sp. strain ER1 KY449279 from estuarine environment. Although Actinomycetes are well known notable producers of several highly active secondary metabolites, their potential is not fully exploited for hydrolytic enzyme production. This study was centered on identifying important fermentation conditions affecting xylanase production using beechwood xylan as the substrate. The use of optimized medium, after considering thirteen factors screened, resulted in a 1.56-fold increased level of the xylanase [93].

### 3.9. Ulvan

The term “ulvan” is used as a common name for sulfated polysaccharides from members of the green algae Ulvales, mainly *Ulva* species. Ulvans are composed of glucuronic acid (or its C-5 epimer, iduronic acid) and α-l-rhamnose sulfated at 3-O position. The structure of the main repeating disaccharide in ulvan, ulvanobiuronic acid, is characterized by β-1,4 linkage of glucuronic acid to rhamnose. Large differences detected in ^13^C-NMR spectra of ulvans isolated from different species of *Ulva* and *Enteromorpha* also indicate the great variability of these polysaccharides, which may also contain different amounts of xylose [94].

Ulvans are of interest for food, pharmaceutical, agricultural, and chemical applications in particular for their physicochemical properties and for biological antitumor, anticoagulant and antiviral actions [95].

Ulvan lyase cleaves the linkages between rhamnose 3-sulfate and glucuronic acid giving rise to oligosaccharides carrying an unsaturated uronic acid residue at the nonreducing end. Other enzymes are usually requested for full saccharification of the polymers such as β-glucuronyl hydrolase, rhamnosidase, xylosidase and sulfatase activities.

Polysaccharide utilization loci (PULs) are a set of co-regulated genes that have been predicted in numerous marine microorganisms. *Alteromonas* species have been recently sequenced in search of new polysaccharide lyase and of a PUL encompassing putative β-glucuronyl hydrolase, sulfatase and rhamnosidase potentially involved in the degradation. In particular, a novel ulvan lyase, unrelated to all the previously reported enzymes, is proposed as a founding member of a new polysaccharide lyase family [96].

### 3.10. Cellulose

Cellulose is the most common polysaccharide, simply consisting of β-(1-4) linked d-glucose units. Along with chitin it represents the most abundant polysaccharide in the marine environment followed by agar, alginates, and carrageenans. For its particular importance this paragraph has been reserved only to cellulose while laminarin and other general β-glucans are discussed in the paragraph above.

According to phylogeny of the algae it is known that the mapping of constituents of cell wall is an important aspect. Some polysaccharides are ubiquitous, however subtleties in structures linked to phylogeny are of interest; cellulose is one of these cases specifically regarding microfibril diameters, that control the physical properties, and enzymes involved in their assembling; however, it is out of the scope of this article to go in depth on macromolecular organization of cellulose.

Many uses of cellulose and its derivatives (carboxymethyl cellulose) are known in the paper, pharmaceutical, textile, veterinary, food and cosmetic industries. Hydrolysis of cellulose to the reducing sugars by enzymatic methodologies has great potential value in many industries such as food, animal feed, bioenergy, textiles, detergents, etc.

Cellulases are complex enzymes presenting exoglucanase, endoglucanase and β-glucosidase activities. Their synergistic action is at the base of enzymatic hydrolysis of lignocellulosic biomass. The first two (endoglucanase and exoglucanase) have the function of depolymerization of cellulose. These activities seem to be scarcely expressed in marine originating *Aspergillus niger* used in processing cellulosic raw material from high salt enviroments (marine waste, marine algae, etc.). One gene of endoglucanase/exoglucanase in *Piromyces rhizinflata* cellulase has been expressed in *A. niger* in order to remarkably increase endoglucanase and exoglucanase activities [97]. The same group studied halostable cellulase from a marine *A. niger* isolated from East China Sea [98]. Particularly they also found exoglucanase activity. Secondary structure changes caused by high salinities improved thermostability and activity for this enzyme [99,100].

Other marine fungi were studied as potential microbial producers of cellulases, *Penicillium citrinum* CBMAI 1186, *Aspergillus sydowii* CBMAI 934, *Aspergillus sydowii* CBMAI 935 and *Mucor racemosus* CBMAI 847. Their cultivation parameters were recently studied in solid state fermentation for producing cellulases. These marine fungi exhibit significant potential for the production of fermentable sugars from cellulose of sugarcane bagasse after pretreatment with NaOH solution [101].

Actinobacteria are economically and biotechnologically of interest for their ability in metabolizing many different compounds. A screening for cellulases in a marine actinobacterial strains from the sediments of Havelock Island in the Indian Ocean has also been reported. Four strains, out of the 19 analyzed, possessed good cellulose degradation activity. The best producer belongs to the genus *Actinoalloteichus* [102].

An important aspect related to carbohydrate active hydrolases in biofuel applications is the co-production of efficient enzymes in one organism. Alginate lyases and cellulases co-produced by a microbial strain are of industrial demand for bioprocessing cost efficiency; crude enzymes can be also used directly. During a bioprospecting investigation of alginolytic and cellulolytic bacteria, an invasive seaweed belonging to *Sargassum* species from Barbados’ coast were found to host a bacterium, identified as *Exiguobacterium* sp. Alg-S5, that simultaneously produces both enzymes. Being alginate and cellulose constituting between 10–40% and 6–20%, respectively of the dry weight of brown algae, the potential industrial importance of this bacterium in this algal biomass conversion was highlighted [103].

The green seaweed *Ulva fasciata* was selected as a potential feedstock for cellulose production for synthesis of sodium carboxymethyl cellulose (CMC). Possessing high growth rate, this species is easy to harvest and process. CMC characterization has been recently published. The properties of films prepared using this marine originating carboxymethyl cellulose match well with the commercial CMC. Marine CMC antimicrobial and biodegradable nature is of interest for various industries [104].

Another interesting producer of marine cellulases is *Streptomyces parvulus* strain sankarensis-A10, possessing many other interesting enzyme activities, found among twelve actinomycetes isolated from a marine sediment sample [105]. Interesting enzymes of this family are also detected during the study of underexplored alga-associated cultivable microbiota [31].

### 3.11. Mannan

β-1,4-Mannan is a major component of hemicellulose found in higher plants and red algae. It consists of β-1,4-linked d-mannopyranose residues. Endo-β-1,4-mannanase (β-mannanase, EC 3.2.1.78) is the enzyme that catalyzes the endolytic hydrolysis of β-1,4-mannosidic linkages in mannopolysaccharides such as β-1,4-mannan, glucomannan, and galactomannan. Mannosidases are also important enzymes in this respect. The most recent original article mentioning marine enzymes belonging to this class is related to a mannanase and dated 2006, thus it is out of the time limit range of this review; the enzyme originated from Pacific abalone *Haliotis discushannai* [106].

### 3.12. Pectin

Pectins are biopolymers whose main structural feature is the linear (1-4)-linked chain of galacturonic units. Very few modern reports are found for marine originating pectins. An article on a pectin like polysaccharide from an edible seaweed *Zostera caespitosa* Miki [107] was found. However, pectins from sea grasses are considered as promising substances with pronounced metal binding activity because of their 3D structure [108].

Pectate lyase is the enzyme of the pectinase group that exhibits a β-elimination mechanism in the cleavage of α-1,4-glycosidic bonds of polygalacturonic acid producing unsaturated Δ-4,5 bond at the nonreducing end of the polysaccharide generating 4,5-unsaturated oligogalacturonates and CO_2_, via E2 elimination mechanism. No original articles on this enzyme were found in searching for marine pectinases or pectin lyases. Marine pectin esterase as keyword found only two review articles and two book chapters.

## 4. Patents

While searching the literature for the previous general survey on enzymatic processes in marine biotechnology [8], different interesting aspects were noticed; two time intervals were recognized for the hits sorted with two different global average amount of articles per year; a doubling value was found yearly from 2012 to 2016 with respect to that for the time interval 1993 to 2011 characterized by fewer than 20 hits per year. This picture was interpreted as probably reflecting the strategic efforts of various funded programs created by the European Commission to support and foster research in the European Research Area and similar actions in other countries. Going into details, only half of the hits found were original research articles, being most of the remaining ones represented by books or reference works. It is possible that the literature is still showing only a partial result of different governmental boosts to research and more time is needed to appreciate in full the complete output of supporting this research. Indeed, it is hopeful that early grand results are at the moment pre-commercialization reports, patents etc. with primary applicative aim. Patents are in fact a valuable font of technical knowledge showing the current or past state of technology and fully presenting the whole potential of a scientific topic. These hits are usually neglected in a normal literature search, not analyzed in [8]. Appearance of high quantity of reference works cited above could be acting as a mask/substitute for them.

A more complete picture and precise answers would be obtained if patents were included in the analysis. Therefore, the same search terms used for Science Direct database have also been adopted for a patent search using the Espacenet patent database.

Table 1 lists the results of such investigation related to 2016–2017 along with title of the hit and year of publication. The choice limited to two years as for original articles is dictated by comparison needs and the results do not represent a general analysis of patenting in the field. Furthermore by close analysis only patents including use of marine enzymes are selected avoiding to include hits in which the word marine is related only to biomasses.

Two patents are present for agarases, the first where the activity is included in an enzyme complex with other biocatalysts for an efficient conversion of marine algae biomass and the other for industrial enzyme production from a microorganism derived from a marine organism. Chitinases were involved in a patented method (enzymatic pre-treatment) for marine phospholipids extraction, obtaining high-purity compounds and in a technique for measuring ecosystem pollution (KR20170027395 and KR20170027393). As for amylases: (i) a patent related to a novel marine psychrophilic producer, *Colwellia* sp. applied to produce, among others, this kind of enzymatic activity by liquid fermentation, was found and (ii) a rapid and efficient expression of a low-temperature marine amylase gene is also implemented allowing production of a great quantity of marine low-temperature amylase. Among inventions found, a marine glucanase from *Bacillus marinus* was reported; high activity and heat-pH stability characterizes this enzyme with wide potential and application prospects in various industry sectors (CN106011113, CN105695434). Alginate lyase is mentioned in a patent related to a fertilizer composed of 20–50% of seaweed extract subjected to enzymatic pretreatment by the enzyme. A marine bacterial κ-carrageenase gene was mentioned in a patent, it was engineered in *E. coli* putting the base for industrial production of the enzyme. Production by fermentation was improved by 6.46 times for the κ-carrageenase activity of *Thalassospira* sp. Fjfst-332 as described in an invention related to a combined inducer based on kappa gum.

## 5. Conclusions and Perspectives

Figure 1 above provides a pictorial summary of the current research topics/fields on specific polymers as indicated in paragraphs in this review, from the most important reference works and original articles found here. The interdisciplinary nature of the current interest in marine polysaccharides and marine carbohydrate-hydrolyzing enzymes is evident. A number of interesting original articles are found in the active research field related to bioprospecting marine agarases, marine chitinases and xylanases probably reflecting the ongoing successes in the understanding their machinery for bond cleavage and more and more oriented practical applications, especially in the biofuel domain or for the production of small oligosaccharides of pharmaceutical interest. Among biochemical characteristics worth noting, operativity under alkaline condition and solvent stability are the most appreciated.

Alginates and their processing marine enzymes are another big area where research seems to be very active during the last years. Alginate degradation metabolism has been studied in details ad recombinant alginate lyases are in the hand of scientists ready for applications.

Alltogether, it seems that the biocatalysts acting on various marine only-glucose polymers are of great importance. Extremophilic amylases are also interesting as model biocatalysts to assess the molecular basis of their biochemical resistance. Oligosaccharides of the laminarin type and their production are catching attention for their applications in food. Cellulases and other glucanases from marine fungi and actinobacteria are of great interest in biofuel applications. 

Fucoidans are intensely studied, widely profiling their presence and structural details; natural functions of these sulfated glycans are also of great interest. Not only enzymes acting of fucosidic linkages are important but also others acting on decorations (deacetylation, desulfation, etc.) deserve attention. It is important here to add a comment about a very recent insightful review depicting not only biomedical properties of fucose-containing sulfated polysaccharides and brown cell wall organization but also listing six research challenges within this field. The need for preparation and structural elucidation of homogeneous fractions is well recognized and among others: comprehensive screening technology, identification of new enzymes for fine structural investigation, standardization of extraction and purification methods [109].

High efficiency in preparation of carrageenan oligosaccharides including the possibility of using immobilized biocatalysts is a topic of great interest for the related enzymes while other specifically distributed polymers are less represented in original articles found in this literature search; few articles are found for ulvan-, mannan- and pectin-hydrolyzing biocatalysts.

This activity-based distribution can be also individuated among the few patents found in the two-year interval time showing that appropriate time is needed from funding research efforts to practical applications.

Scientific perspectives on these biocatalysts can be inferred from the analysis of original articles and as visually summarized in Figure 1. Indeed very recent interesting articles are also added in this final part.

The adhesion of bacterial cells to surfaces is a phenomenon involving polysaccharides and other substances in the EPS matrix with formation of biofilm. This impacts in economical terms for the potential colonization of man-made surfaces in the so called biofouling. A very recent survey on these aspects with particular importance to the roles of marine polysaccharides is in due course of publication [110].

The importance of reporting on hydrolytic enzymes present in “polysaccharide utilizing loci” (PUL) has been already indicated for putative β-glucuronyl hydrolase, sulfatase and rhamnosidase in Alteromonas [96]. This is an interesting aspect that follow up on the article by Wargacki et al. in 2012 [111] about the discovery of a 36–kilo–base pair DNA fragment from *Vibrio splendidus* encoding enzymes for alginate transport and metabolism. Recent articles on this basic research aspect in bioprospecting should also be mentioned here as perspective in the field: (i) the genome of the strain HZ22 of *Algibacter alginolytica* harbors 3371 coding sequences of many carbohydrate-active enzymes (104 glycoside hydrolases and 18 polysaccharide lyases); seventeen polysaccharide utilization loci are predicted to be specific for marine polysaccharides revealing that this strain has good potential for degration of algal polysaccharides [112]; (ii) the complex genetic structure for carrageenan catabolism in the marine heterotrophic bacterium *Zobellia galactanivorans* [113], and the characterization of polysaccharide utilization systems of the marine *Gramella flava* JLT2011 [114] that expands knowledge on the diversity and function of PULs in marine Bacteroidetes.

Probably the complex nature of the pipeline in this scientific field that starts with bioprospecting, using new-related technologies and go through biochemical assessment of enzyme activity, strictly related to the chemical complexity of their substrates, up to generating engineered platforms for polysaccharide metabolization, justifies the massive presence of reference material as a signal of scientific (and editorial) interest.

## Figures and Tables

**Table 1:** Patents found for marine carbohydrate hydrolyzing enzymes for 2016–2017 interval time in Espacenet patent database.

Keyword	Publication	Title	Year
marine agarase	W02017111208(1)	Enzyme complex comprising beta agarase, kappa carrageenase and anhydrogalactosidase, and use thereof	2017
	CN105420142 (A)	Bacterium *Pseudoalteromonas*.sp.QJ97 derived from marine organisms and method for producing agarase through bacterium *Pseudoalteromonas* sp. QJ97	2016
marine chitinase	CN106588977 (A)	Method for extracting high-purity marine phospholipids from *Euphausia superba*	2016
	KR20170027395 (A)	Measurement apparatus of marine environment pollution indicator using a surface roughness of *Macrophthalmus japonicus*	2017
	KR20170027393 (A)	Irgarol 1051 method of chitinase gene mRNA analysis of *Macrophthalmus japonicus* toxic according to Irgarol 1051	2017
marine amylase	CN106754501 (A)	Novel psychrophilic strain of *Colwellia* sp. as well as culture method and application thereof	2017
	CN106148367 (A)	Clone of marine low-temperature alpha-amylase gene and expression thereof	2016
marine laminarinase marine glucanase	CN106011113 (A)	Beta-glucanase generated by *Bacillus marinus* and preparation method thereof	2016
	CN105695434 (A)	Novel beta-glucanase and preparing method thereof	2016
marine alginate lyase	CN106220364 (A)	Composite leaf fertilizer and preparing method and application thereof	2016
marine carrageenase	CN105950640 (A)	Preparation method of marine bacteria-derived kappa-carrageenase gene and recombinase	2016
	CN105483100 (A)	Combined inducer inducing marine microbial fermentation to produce κ-carrageenase	2016
